# Effect of integrating a video intervention on parenting practices and related parental self-efficacy regarding health behaviours within the Feel4Diabetes-study in Belgian primary schoolchildren from vulnerable families: A cluster randomized trial

**DOI:** 10.1371/journal.pone.0226131

**Published:** 2019-12-11

**Authors:** Vicky Van Stappen, Sara De Lepeleere, Nele Huys, Julie Latomme, Maïté Verloigne, Greet Cardon, Odysseas Androutsos, Yannis Manios, Ilse De Bourdeaudhuij, Marieke De Craemer

**Affiliations:** 1 Department of Movement and Sports Sciences, Ghent University, Ghent, Belgium; 2 Research Foundation Flanders (FWO), Brussels, Belgium; 3 Department of Public Health and Primary Care, Ghent University, Ghent, Belgium; 4 Department of Nutrition and Dietetics, School of Health Sciences & Education, Harokopio University, Kallithea, Athens, Greece; 5 Department of Rehabilitation Sciences, Ghent University, Ghent, Belgium; University of Auckland, NEW ZEALAND

## Abstract

**Background:**

This study aimed to investigate the effect of integrating a video intervention “Movie Models” within the Feel4Diabetes-study on specific parenting practices and related parental self-efficacy regarding children’s physical activity, screen-time and eating behaviour in vulnerable families (i.e. families living in low socioeconomic municipalities and at risk for developing type 2 diabetes). Additionally, there was examination of how the intervention was perceived by the parents.

**Methods:**

Within randomly selected low socioeconomic municipalities in Belgium, families were recruited through primary schools. Families at risk for developing type 2 diabetes were identified using the FINDRISC questionnaire (n = 457). Afterwards, the municipalities were randomly assigned to the intervention or control condition. At risk families assigned to the intervention group were invited to participate in six Feel4Diabetes counselling sessions in which families were encouraged to adopt a healthier lifestyle. The “Movie Models” videos were integrated within two sessions by using a face-to-face group discussion approach. Parenting-related factors were assessed before and after the integration of the videos, using a questionnaire. After integrating the videos, some extra evaluation questions were assessed. In total, 126 families were included in a per protocol evaluation and Repeated Measures ANOVAs were conducted to evaluate the potential intervention effects.

**Results:**

Some favourable intervention effects were found on parenting practices and related parental self-efficacy regarding children’s eating behaviours, however almost no effects were found on parenting-related factors regarding children’s physical activity and screen-time. In total, 60.0% of the participants indicated that they applied tips regarding parenting practices and 52.0% indicated that discussions with other participants regarding the videos were useful for them.

**Conclusion:**

The integration of “Movie Models” within the Feel4Diabetes-study was effective in improving some parenting-related factors regarding children’s health behaviours, however most parenting-related factors could not be improved. The implementation of “Movie Models” as a face-to-face group discussion approach was relatively well received and may be a promising way to improve parenting-related factors in vulnerable families.

**Trial registration:**

ClinicalTrials.gov NCT02278809.

## Background

In Europe, the prevalence of overweight and obesity has become a public health problem, even at a young age [1]. A European study conducted in eight countries indicated that 7.0% of the children below the age of 10 were obese and 12.8% overweight [2]. In addition, research indicated that childhood obesity tracks into adulthood [3], which may result in negative health consequences [4], including type 2 diabetes (T2D). Overweight and obesity are responsible for about 80% of the cases of T2D among adults in the European region [4]. To tackle T2D and other health problems associated with overweight and obesity in adulthood, it is important to develop effective overweight and obesity prevention programmes for young children.

Physical activity (PA), screen-time and eating behaviour have a considerable impact on children’s weight status [5–7]. Movement guidelines for children (aged 5–17 years) recommend to engage in at least 60 minutes of moderate-to-vigorous PA (MVPA) per day [8] and to not exceed two hours of recreational screen-time per day [9, 10]. However, results from the European IDEFICS-project, based on objective measures, showed that only between 2.0% (Cyprus) and 34.1% (Belgium) of the children between 2 and 11 years old met the recommended 60 minutes of MVPA per day [11]. Furthermore, 25.0% of the girls and 33.0% of the boys exceeded the recommended daily screen-time limits [12]. In addition, guidelines formulated by the World Health Organization (WHO) regarding the daily food consumption of children between 6 and 11 years recommend to consume at least 400 grams of fruit and vegetables [13], about 1.5 litres of water per day [14], and to limit the daily consumption of unhealthy snacks and soft drinks [15]. Research has indicated that between 7.8% and 24.1% of 11-year-old European children did not reach the WHO goals regarding fruit and vegetable intake [16]. Moreover, among 6- to 14-year-old Belgian children, only 29.0% consumed the recommended amount of water per day, 55.0% consumed unhealthy sweet or salty snacks on a daily basis and 26.8% consumed at least one soft drink a day [17]. Last, recent studies have indicated that unhealthy behaviours are generally more likely to occur in vulnerable families (e.g. low socioeconomic status (SES) families, families with overweight and obese children) [17–21]. Taken together, there is a need to develop effective interventions to improve young children’s health behaviours, especially among vulnerable families.

Parents play an important role in developing and shaping children’s health behaviours [22, 23]. Parenting practices (e.g. motivating your child to be physically active) refer to content- and context-specific childrearing approaches that parents use to bring certain childrearing outcomes (e.g. the engagement of children in PA) [24]. Recent research has indicated that low educated parents (≤14years of education) are less likely to follow favourable parenting practices (e.g. avoiding negative modelling) related to eating behaviour compared to higher educated parents [25], suggesting there is a need for interventions targeting those practices in an at-risk group. Interventions targeting parenting practices have shown to be an effective strategy for the prevention and management of childhood obesity [26]. However, interventions targeting parenting practices in vulnerable groups are scarce. Only a few studies have already examined the effectiveness of an intervention targeting parenting practices in a vulnerable population. In most studies, the focus was on families with overweight and obese children [27–29], however to the best of our knowledge only one intervention (i.e. The Healthy Children, Healthy Families: Parents Making a Difference! Intervention) was developed to encourage low-income parents to adopt parental strategies to facilitate progress on the path to health behaviours at home. Results showed significant improvements in several outcomes of both the parents and child (e.g. improvements in children‘s fruit intake and screen-time, improvements in parenting practices related to family meals) [30–32]. Furthermore, enhancing parental self-efficacy concerning parenting practices (i.e. the expectation parents hold about their ability to perform positive parenting practices) might be an important step in effectively adopting these parenting practices [33]. Therefore, it would be relevant to assess the effect of an intervention on parental self-efficacy concerning parenting practices as well, however, no previous intervention in vulnerable populations have investigated the effect on such outcome.

Recent studies indicated that narrative approaches (such as entertainment education, storytelling) are a promising set of tools for motivating and supporting health-behaviour change. Narratives can influence health-behaviour change by behavioural modelling or observational learning. These mechanisms are based on the social cognitive theory, which states that by observing a model, individuals can learn a specific behaviour (e.g. parental practices) and they will be more likely to perform it [34]. Additionally, research in Flanders (Belgium) in which focus groups with parents and discussions with stakeholders and parenting experts were held, highlighted an interest in videos as a way to learn how to perform effective parenting practices [35]. Therefore, the use of video-based narratives as an intervention strategy is a promising approach to increase parenting-related factors.

The aim of the current study was to investigate the effect of a video intervention “Movie Models” on parenting practices and related parental self-efficacy regarding PA, screen-time and eating behaviour in children from vulnerable families (i.e. families living in low SES municipalities and with an increased risk for developing T2D). Besides evaluating the effect of the intervention, it is important to examine participants’ satisfaction/perception of the intervention in order to better understand the potential effects of a health promotion intervention, to predict attrition and to determine intervention aspects to improve [36–38]. Therefore, an additional was to study how parents perceived the intervention, by using questionnaire data.

### Methods

In Belgium, the “Movie Models” intervention was integrated within the European Feel4Diabetes-intervention and was implemented between September and March 2017. Below, more information is provided about the aim and content of both interventions and how the “Movie Models” intervention was integrated. The Feel4Diabetes-intervention was registered at https://clinicaltrials.gov/ (registration number: NCT02393872).

#### Feel4Diabetes-intervention

Within the Feel4Diabetes-study, a school- and community-based, family-involved intervention was developed aiming to promote a healthy lifestyle and tackle obesity-related metabolic risk factors for the prevention of T2D in families living in low SES municipalities across six European countries (Belgium, Bulgaria, Finland, Greece, Hungary and Spain). The Feel4Diabetes-intervention focused on three components: (1) the high-risk families component (focusing on families with an increased risk for developing T2D based on the diabetes risk score–see Methods section, Measurements, Diabetes risk score) (2) the school component and (3) the community component. More detailed information about the development and the content can be found elsewhere [39]. As parenting practices and related parental self-efficacy were mainly addressed at the high-risk families component, we will elaborate on this specific component of the Feel4Diabetes-intervention. In the high-risk families component, families assigned to the intervention and control group received a folder with tips and recommendations regarding a healthy lifestyle and feedback on their anthropometric measurements and blood results. In addition, families assigned to the intervention group were invited to participate in six counselling sessions (including one individual session and five group sessions) in total, of which five sessions were delivered between September 2016 and March 2017 and the sixth session was delivered in September 2017. During the counselling sessions, delivered by trained health professionals, families were encouraged to adopt a healthier and more active lifestyle, with a focus on more PA, less sedentary behaviour (SB), and healthy eating behaviour. The Feel4Diabetes-intervention was developed jointly for the six countries, but each country could make local adaptations to local needs and contextual circumstances.

#### “Movie Models” intervention

“Movie Models” is a video intervention, aiming to improve children’s health behaviours (i.e. PA, screen-time and eating behaviour) by addressing parenting practices and parental self-efficacy concerning parenting practices related to these behaviours. The “Movie Models” intervention is based on the principles of the Self Determination Theory and developed according to the Intervention Mapping Protocol [40]. In the “Movie Models” intervention 22 videos (each lasted about 2 minutes) were developed in which a difficult child-parent situation is presented and followed by an appropriate reaction of the parents. Afterwards, a narrator explains the parenting practices used in the video, so parents can learn effective parenting strategies throughout the modelling technique and furthermore, parental knowledge, attitude and self-efficacy concerning these parenting practices could be enhanced. More information about the development and the content can be found elsewhere [40]. The pilot study of “Movie Models” has been previously tested as an online video intervention in which a convenience sample of parents, recruited via schools and (social) media, were invited to watch the parenting videos on a secured website. Unfortunately, results only showed limited favourable intervention effects. This may be attributed to the intervention that reached predominantly high SES parents with children with a normal Body Mass Index (BMI), resulting in high baseline values for the parenting practices and related parental self-efficacy and possible ceiling effects [41]. Therefore, the researchers recommended to test the “Movie Models” videos in vulnerable parents, who may have poorer parenting practices and less parental self-efficacy concerning parenting practices related to health behaviours [41].

#### Integration of the “Movie Models” videos within the Feel4Diabetes-intervention

**Fig 1:**
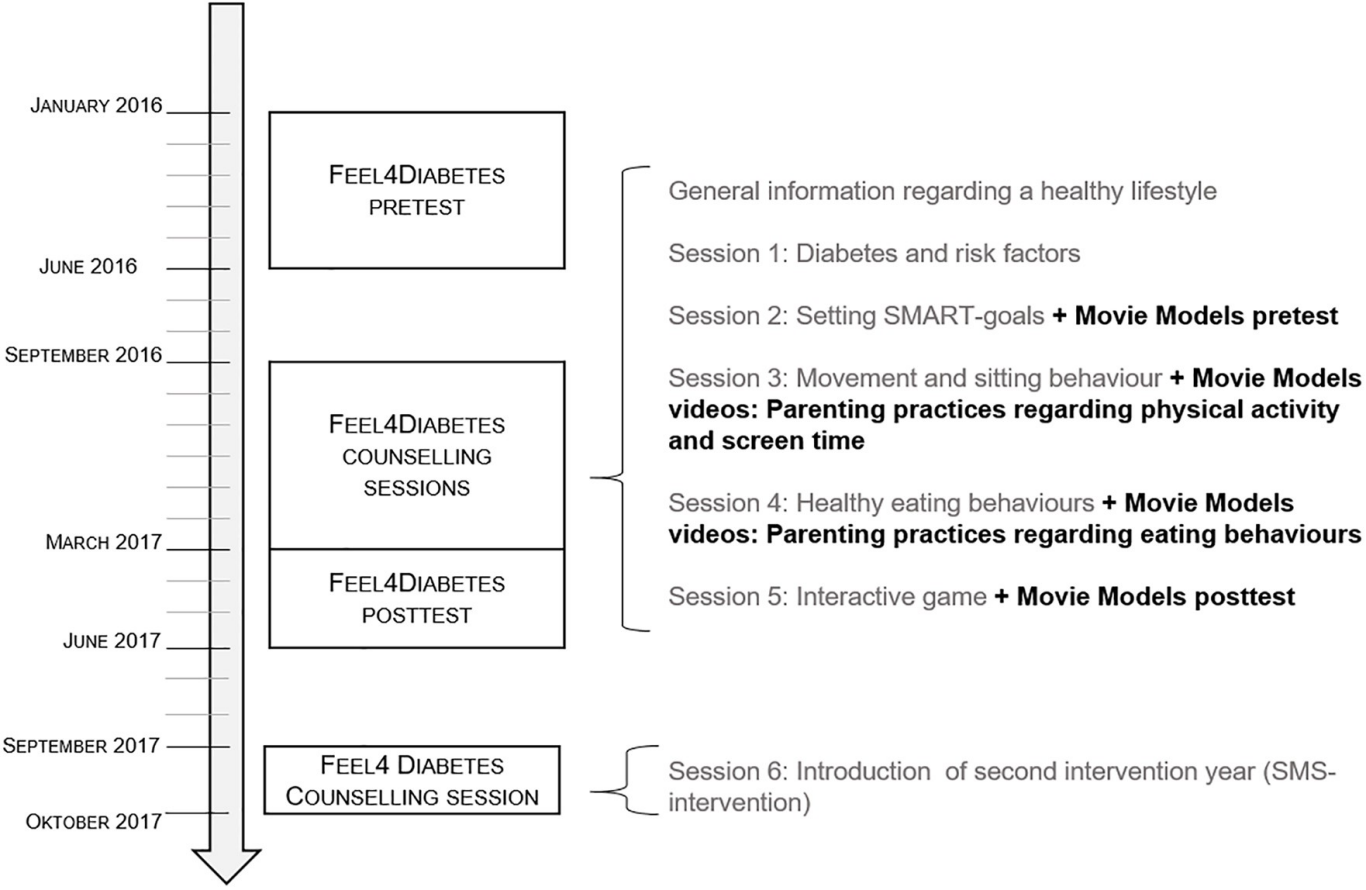
Integration of “Movie Models” intervention within the Belgian cohort of the Feel4Diabetes-intervention.

Within the Feel4Diabetes-intervention, some country-specific adaptations were made. In Belgium, the “Movie Models” videos were integrated within the Feel4Diabetes-intervention, as targeting parenting practices have shown to be an effective strategy for the prevention and management of childhood obesity [26]. The “Movie Models” videos only exist in Dutch, so it was not possible to integrate the videos in the other countries. Thirteen out of the 22 “Movie Models” videos fitted within the themes of the Feel4Diabetes counselling sessions and were integrated within two Feel4Diabetes counselling sessions, namely six videos were integrated in the counselling session on PA and SB and seven videos in the counselling session on healthy eating behaviour. Five videos regarding guidelines of health behaviours were excluded because these guidelines were already integrated within the Feel4Diabetes-intervention in another way and four videos were not integrated because of the limited added value and to limit the duration of the counselling sessions. In contrast to the first “Movie Models” pilot study (in which the videos were available online and participants were asked to watch them individually at home), the videos were shown to the participants in group and were discussed afterwards (i.e. a face-to-face group discussion approach). Then, all intervention parents that participated in the high-risk families component of the Feel4Diabetes-intervention (i.e. both parents who attended those sessions including the “Movie Models” intervention and parents who did not attend those) received a link to the webpage of the videos by e-mail. Specific parenting practices and related parental self-efficacy regarding children’s health behaviours were assessed during the Feel4Diabetes intervention period (“Movie Models” pre-test: November-December 2016 and post-test: February-March 2017). Furthermore, parents were asked to fill out a questionnaire regarding their perception of the intervention (February-March 2017). Fig 1 shows an overview of the integration of the “Movie Models” videos within the Feel4Diabetes-intervention and an overview of the content of the integrated “Movie Models” videos within the Feel4Diabetes-intervention is provided in Table 1.

**Table 1:** The “Movie Models” videos as part of the Feel4Diabetes-intervention.

**Group session 4: “Movie Models” videos, parenting practices regarding physical activity and screen-time**
“Movie Models” videos: parenting practices regarding *physical activity*
*Video 1*: *Try to be active in as many ways as possible during the day*
*Video 2*: *Be a good role model for your child by being physically active together with him/her*
*Video 3*: *Make physical activities pleasant for your child*
“Movie Models” videos: parenting practices regarding screen-time
*Video 4*: *Enter into agreements about screen-time*, *and be consistent*
*Video 5*: *Do not use TV or computer as a mean to keep your child calm*
*Video 6*: *Do pleasant activities together with your child instead of watching TV*
**Group session 5: “Movie Models” videos, parenting practices regarding eating behaviour**
“Movie Models” videos: parenting practices regarding water consumption
*Video 7*: *Let your child drink water limitless*, *and make rules concerning soft drink consumption*
*Video 8*: *Motivate your child to drink water*
“Movie Models” videos: parenting practices regarding fruit intake
*Video 9*: *Reinforce your child when he/she eats fruit*
“Movie Models” videos: parenting practices regarding vegetable intake
*Video 10*: *Motivate your child each time to taste a vegetable he/she does not like*
“Movie Models” videos: parenting practices regarding breakfast
*Video 11*: *Make sure breakfast is a peaceful and calm moment*
“Movie Models” videos: parenting practices related to grocery shopping
*Video 12*. *Involve your child in buying fruit and vegetables in the supermarket*
*Video 13*: *Limit buying unhealthy food as much as possible*

### Study protocol

**Fig 2:**
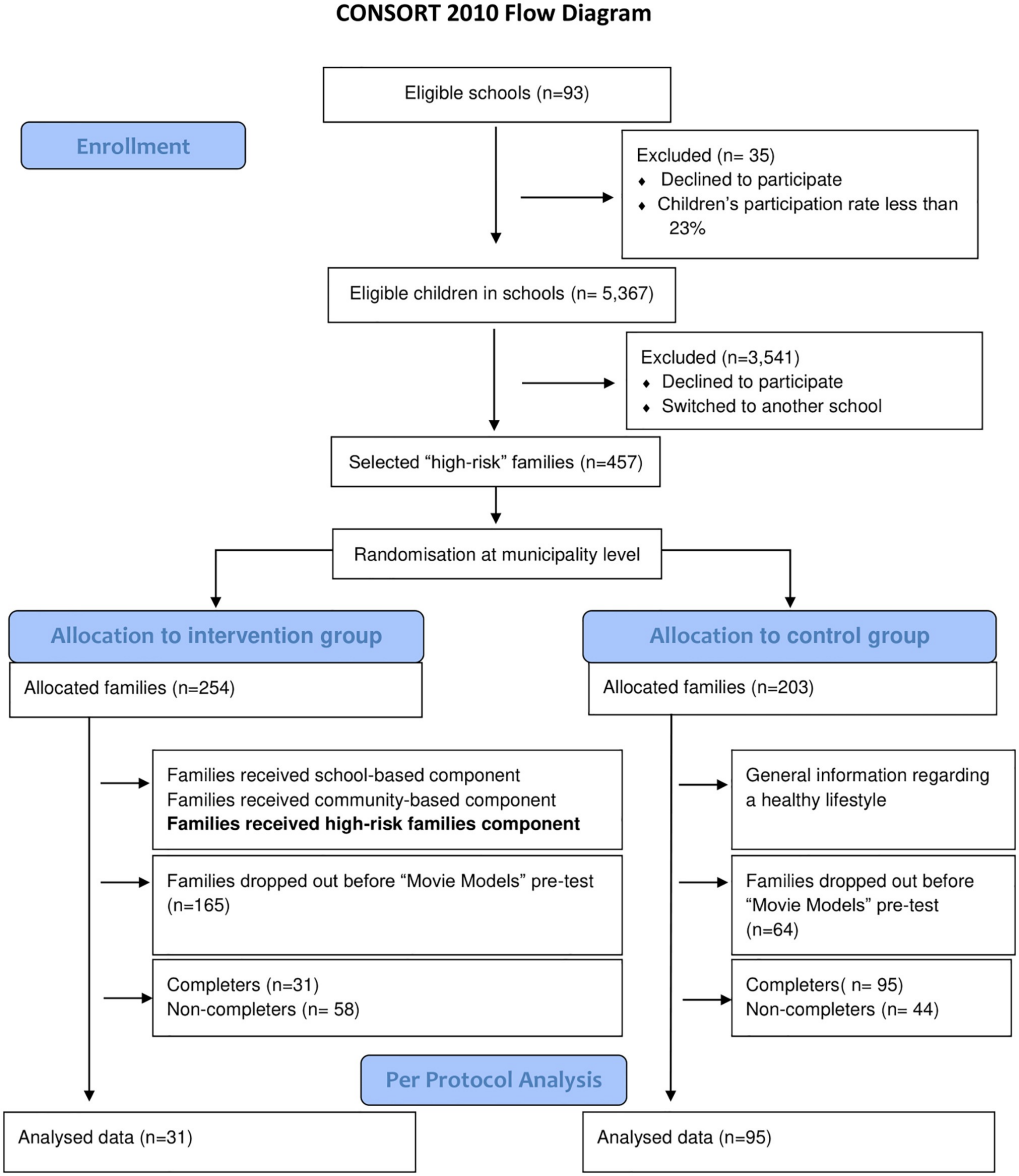
CONSORT Flow Diagram.

In Belgium, all municipalities within a range of 40km around the city centre of Ghent (location of Ghent University) were selected. These municipalities were divided into tertiles based on unemployment rate [42]. Within the highest tertile (municipalities with the highest unemployment rates (range 5.2–12.5%)), 11 municipalities were randomly included in the study. Furthermore, schools (n = 93) within those 11 municipalities were randomly contacted and the head masters of 58 primary schools (response rate = 68.8%) confirmed their participation in the Feel4Diabetes-study. All parents of primary schoolchildren from the first, second and third grade (aged 6–9 years) received a participant information sheet, a consent form, the FINDRISC-Questionnaire (Finnish Diabetes Risk Score-Questionnaire; a tool that assesses the 10-year risk of developing T2D [43]) and the Energy-Balanced Related Behaviours Questionnaire (EBRB-Questionnaire). In total 1,572 parents (response rate = 34.0%) consented to the participation of their family (child and at least one parent) by signing the consent form and filling out both questionnaires (March-June 2016). In total 457 families (29.1% of the participants) were identified as families with an increased risk for developing T2D (based on the diabetes risk score–see Methods section–Measurements–Diabetes risk score). Next, municipalities were randomly assigned to the intervention or control condition (i.e. cluster randomised control trial), so the randomisation did not occur at the individual level. In total, 254 families were assigned to the intervention group and 203 families to the control group. Throughout the study many participants dropped out for several reasons (e.g. no time, not interested, health problems, child changed schools). Before the “Movie Models” pre-test 165 intervention families (65% of the families allocated to the intervention group) and 64 control families (32% of the families allocated to the intervention group) dropped out. Fig 2 shows the recruitment procedure and the flow of the participants through the study. Ethical approval was granted by the Ethical Committee of the Ghent University Hospital (Belgium), B67021214212 (“Movie Models”) and B670201526922 (Feel4Diabetes).

### Measurements

Individuals risk for developing T2D, specific parenting practices related to children’s health behaviours, parental self-efficacy concerning these parenting practices, socio-demographic variables and data regarding parents’ perception of the intervention were obtained by questionnaires.

#### Diabetes risk score

The FINDRISC-Questionnaire is a validated screening tool for predicting one’s risk on T2D, including eight questions on age, BMI, waist circumference, PA, daily consumption of fruit, berries or vegetables, history of antihypertensive drug treatment, history of high blood glucose and family history of diabetes [44, 45]. Because a FINDRISC-score of at least 9 points identifies more than 70% of incident cases of T2D, this score is often used to identify individuals at risk for developing T2D [46]. Because the Feel4Diabetes-study aimed to target a healthy lifestyle and tackle obesity-related metabolic risk factors, additional criteria were used in Belgium for identifying individuals at risk for developing T2D. In Belgium, families were assigned to the high risk group if at least one of the parents had a FINDRISC-score of ≥9 and additionally either an unhealthy lifestyle (not eating fruit and vegetables every day and not being sufficiently physically active every day) or an increased waist circumference >102cm (men) and >88cm (women), which indicates a risk of metabolic complications [47].

#### Primary outcomes

In total 62 specific parenting practices were assessed using a newly developed scale based on existing and validated questionnaires: (1) the Parental Support For Physical Activity Scale (Cronbach’s alpha = 0.78; test-retest reliability: R = 0.81) [48], (2) the Parenting Strategies for Eating and Activity Scale (Cronbach’s alpha = 0.81–0.82) [49] and (3) the Parental Feeding Style Questionnaire (Cronbach’s alpha = 0.67–0.83; test-retest reliability: R = 0.76–0.83) [50]. Parenting practices were measured using a 5-point Likert scale: 1 = Never, 2 = Rarely, 3 = Sometimes, 4 = Often, 5 = Always (for example a score of 5 indicates that the parent “always” applied the specific parental skill). Furthermore, 57 parental self-efficacy questions were based on the validated GEMS questionnaire (Cronbach’s alpha: 0.52–0.62, test-retest reliability: R = 0.61–0.82), the questionnaire of parental self-efficacy for enacting physical activity and healthy diet in their children (Cronbach’s alpha: 0.94; test-retest reliability: R = 0.94) [51] and Section L of the Aventuras Para Ninos parent survey (Cronbach’s alpha: 0.73–0.87) [52]. Parental self-efficacy concerning parenting practices were measured using a 5-point Likert scale: 1 = Completely disagree, 2 = Mostly disagree, 3 = Sometimes disagree/ Sometimes agree 4 = Mostly agree, 5 = Completely agree (for example a score of 4 indicates that the parent mostly agreed that they can be successful in applying specific parental skills). All variables about parenting practices and related parental self-efficacy can be found in Supporting file 1 (S1 Table). For all variables, a higher mean value represents a higher form of the variable.

#### Socio-demographic variables

Age and sex of the participating child and parents’ age, sex and educational level were obtained from the EBRB-questionnaire. Both, area level measures and education were used as a proxy of SES. Families were recruited within low SES municipalities which makes them vulnerable for developing T2D [53]. Furthermore, low family SES was defined as both parents having no higher education (≤14 years of education). Medium family SES was defined as at least one of the parents having no higher education, and a high family SES was defined as both parents having a higher education (>14 years of education). In European education systems more than 14 years of education implies attendance of higher education (e.g., bachelor program). Socio-demographic variables were assessed at Feel4Diabetes pre-test (January-June 2016). Finally, to determine children’s and parents’ BMI (kg/m^2^), their height and weight was objectively measured respectively at school and at home (April and June 2016). Children and parents with a BMI ≥25 were classified as being overweight or obese.

#### Parent’s perception of the intervention

To evaluate how the integration of the “Movie Models” videos within the Feel4Diabetes-intervention was perceived by the parents, a questionnaire was developed. The perception of the “Movie Models” videos was assessed making use of the following six statements: (1) “The videos about parenting practices were an important part of the entire intervention”, (2) “I apply tips regarding parenting practices that are covered in the videos within my family”, (3) “The discussion with the other participants regarding the videos on parenting practices were instructive for me”, (4) “The discussion with the other participants regarding the videos on parenting practices ensured that I applied tips within my own family”, (5) “I talked with others (non-participants) about the videos on parenting practices”, (6) “Afterwards, I watched the videos at home again”. All items were measured using a 5-point Likert scale: 1 = Strongly disagree, 2 = Rather disagree, 3 = Neither agree or disagree, 4 = Rather agree, 5 = Strongly agree. Answers were recoded into 0 (strongly disagree, rather disagree, and neither agree or disagree) and 1 (rather agree and strongly agree).

### Statistical analyses

Analyses were conducted in SPSS statistics 24.0 for Windows (SPSS Inc., Chicago, IL). Families were excluded for the analyses if the questionnaire regarding parenting practices and parental self-efficacy concerning parenting practices was filled out by another parent (for example mother on pre-test and father on post-test) or for another child and if participants assigned to the intervention group did not receive the entire “Movie Models” intervention (Per Protocol Analysis). Univariate t-tests and chi-square (χ^2^) tests were conducted to investigate differences in socio-demographic factors between intervention group and control group at baseline. Because no differences could be found in socio-demographic characteristics (i.e. sex of parent and child, mean age of parents and child, BMI of parent and family SES), they were not used as covariates in further analyses. Furthermore, univariate t-tests and chi-square tests were performed to investigate differences in socio-demographic factors between completers (i.e. parents assigned to the control group who filled out the questionnaire on parenting practices and parental self-efficacy on pre-test and post-test, and parents assigned to the intervention group who filled out both questionnaires and received the entire “Movie Models” intervention) and non-completers (i.e. parents assigned to the control group who filled out the questionnaire only on pre-test and parents from the intervention group who filled out the questionnaire only on pre-test or did not received the entire “Movie Models” intervention). To examine intervention effects on parenting practices and parental self-efficacy concerning parenting practices, repeated measures ANOVAs were used. Within the analyses, time (pre–post) was defined as a within factor, and condition (intervention group vs. control group) as a between factor. Interaction effects of Time x Condition were reported for each primary outcome (a total of 119 parenting-related factors). To account for multiple testing, p-values ≤ 0.01 were considered as significant and p-values >0.01 and ≤ 0.05 were considered as borderline significant. In order to quantify the magnitude of the post-intervention difference between intervention and control groups, effect sizes were calculated by using SPSS statistics 24.0 for Windows (SPSS Inc., Chicago, IL). An effect size of 0.3 or lower is considered as small, an effect size between 0.3 and 0.5 is considered as medium and an effect size of 0.5 or higher is considered as large [54].

## Results

### Descriptives

In total, 126 parents (31 intervention and 95 control parents; 82.6% women; mean age: 39.2 ± 4.8 years; 54.8% were overweight/obese) with children between 7 and 10 years old (48.4% girls; mean age: 8.19 ± 0.8 years; 15.1% were overweight/obese) completed the questionnaire at the “Movie Models” pre- and post-test and received the complete “Movie Models” intervention. Furthermore, 79 families did not have complete data at “Movie Models” post-test and 23 families did not received the complete “Movie Models” intervention. Descriptive data showed that 27.8% of the families had a low family SES, 25.4% of the families had a medium family SES, and 46.0% had a high family SES. The FINDRISC-score of the families ranged between 9 and 21, with an average score of 12.1 (± 2.4).

Results of the attrition analyses showed that a higher percentages of parents assigned to the control group completed “Movie Models”. In total 75.4% of the completers were assigned to the control group (χ^2^ = 24.65, p<0.001). For the other socio-demographic factors, no significant differences were found between completers and non-completers. The results of the attrition analysis can be found in Table 2.

**Table 2:** Attrition analysis.

	Completers (n = 126)	Non-completers (n = 102)	Comparison	p-value
Condition			χ^2^ = 24.65	<0.001
Intervention n (%)	31 (24.6)	58 (56.9)		
Control n (%)	95 (75.4)	44 (43.3)		
Gender parent			χ^2^ = 1.95	0.38
Fathers n (%)	23 (18.3)	23 (22.5)		
Mothers n (%)	103 (81.7)	78 (76.5)		
Gender child			χ^2^ = 0.82	0.37
Boys n (%)	65 (51.6)	55 (45.5)		
Girls n (%)	61 (48.4)	46 (54.5)		
SES			χ^2^ = 3.44	0.18
Low family SES n (%)	35 (28.0)	32 (32.3)		
Medium family SES n (%)	32 (25.6)	33 (33.3)		
High family SES n (%)	58 (46.4)	34 (34.3)		
Mean age parent -years- mean (SD)	39.2 (4.8)	40.3 (6.0)	t = -1.49	0.14
Mean age child -years- mean (SD)	8.2 (0.84)	8.2 (1.00)	t = -0.50	0.62

SES socioeconomic status

### Intervention effects on parenting practices and parental self-efficacy concerning parenting practices related to PA and screen-time

Results showed favourable, significant intervention effects on one parenting practice related to PA and screen-time (1 out of 20 or 5% of all surveyed parenting practices related to PA and screen-time). A significant intervention effect was found for “Motivating concerning TV or DVD”: Parents from the intervention group had an increase (intervention +0.55 and control +1 on a 5-point scale) in motivating their child to watch less TV or DVD compared to no change in the control group. Concerning parental self-efficacy related to parenting practices, one significant intervention effect was found on parental self-efficacy related to PA and screen-time (1 out of 20 or 5% of all surveyed parental self-efficacy items related to PA and screen-time). A borderline significant, unfavourable intervention effect was found for “Self-efficacy on giving an explanation concerning watching TV or DVD”: Parents from the intervention group showed a decrease (intervention -0.33 and control +0.11 on a 5-point scale) in self-efficacy on giving an explanation concerning TV or DVD, compared to an increase in the control group (+0.11 on a 5-point scale).

Results of the significant intervention effects (mean values and the 95% confidence interval (CI), mean differences between groups and the 95% CI, F-values, p-values and effect sizes) are presented in Table 3. The intervention effects (F- and p-values) and mean values of all parenting-related factors regarding children’s PA and screen-time at the pre- and post-test for both groups can be found in Supplementary file 1 (S1 Table).

**Table 3:** Intervention-effects on parenting practices and parental self-efficacy concerning these practices related to PA and screen-time (variables selected at p<0.05).

Outcome variable	Time	Mean IG [95% CI]	Mean CG [95% CI]	Mean difference between groups [95% CI]	Intervention-effect		
					F-value	p-value	ηp^2^
**Parenting practices**							
Motivating concerning TV or DVD	Pre	3.56 [3.22;3.89]	3.60 [3.41;3.79]	0.04 [-0.34;0.43]	8.49	0.004	0.075
	Post	4.11 [3.84;4.38]	3.61 [3.46;3.77]	-0.50 [-0.81;-0.19]			
**Parental self-efficacy**							
SE giving an explanation concerning TV	Pre	3.96 [3.58;4.35]	3.90 [3.68;4.13]	-0.06 [-0.51;0.39]	4.54	0.035	0.041
	Post	3.63 [3.27;3.99]	4.01 [3.81;4.22]	0.38 [-0.3;0.80]			

IG Intervention group; CG Control group; SE Self-efficacy; CI Confidence interval. ηp^2^ effect size; An effect size of 0.3 or lower is considered as small effect size. F-values, p-values and effect sizes were obtained by conducting repeated measures anova.

### Intervention effects on parenting practices and parental self-efficacy concerning parenting practices related to eating behaviour

Four significant or borderline significant intervention effects were found on parenting practices related to eating behaviour (4 out of 42 or 9.5% of all surveyed parenting practices related to eating behaviour). Parents from the intervention group had an increase in “Modelling fruit consumption of their child” (intervention +0.45 and control -0.12 on a 5-point scale), compared to a decrease in the control group. Parents from the intervention group had an increase in “Setting rules concerning juice consumption” (intervention +0.49 and control +0.03 on a 5-point scale) and “Letting their child choose between different kinds of vegetables” (intervention +0.42 and control +0.01 on a 5-point scale) compared to no change in the control group. Further, intervention parents had a decrease (intervention -0.20 and control +0.16 on a 5-point scale) in “Having snacks available at home” compared to an increase in the control group.

Concerning parental self-efficacy related to parenting practices, four significant intervention effects were found on parental self-efficacy related to eating behaviour (4 out of 37 or 10.8% of all surveyed parental self-efficacy items related to eating behaviour). Parents in the intervention group had an increase in ‘Self-efficacy concerning giving their child an explanation concerning soft drinks (intervention +0.25 and control -0.17 on a 5-point scale), in “Self-efficacy concerning the involvement of their child in buying vegetables” (intervention +0.32 and control -0.10 on a 5-point scale) and in ‘Self-efficacy concerning the involvement of their child in buying fruit (intervention +0.28 and control -0.20 on a 5-point scale) compared to a decrease in the control group. Furthermore, a significant increase in “Self-efficacy in motivating their child to eat more fruit (intervention +0.59 and control +0.02 on a 5-point scale) was found in the intervention group compared to no change in the control group.

Results of the significant intervention effects (mean values and the 95% CI, mean differences between groups and the 95% CI, F-values, p-values and effect sizes) are presented in Table 4. The intervention effects (F- and p-values) and mean values of all parenting-related factors regarding children’s PA and screen-time at the pre- and post-test for both groups can be found in Supporting file 1 (S1 Table).

**Table 4:** Intervention-effects on parenting practices and parental self-efficacy concerning these practices related to eating behaviour (variables selected at p-value <0.05).

Outcome variable	Time	Mean IG [95% CI]	Mean CG [95% CI]	Mean difference between groups [95 CI]	Intervention-effect		
					F-value	p-value	ηp^2^
**Parenting practices**							
Rules concerning juice	Pre	3.17 [2.75;3.60]	3.63 [3.39;3.87]	0.45 [-0.04;0.95]	5.22	0.024	0.042
	Post	3.66 [3.19;4.12]	3.44 [3.18;3.70]	-0.22 [-0.75;0.32]			
Availability of snacks	Pre	4.10 [3.80;4.41]	3.98 [3.81;4.15]	-0.13 [-0.48;0.22]	6.99	0.009	0.056
	Post	3.90 [3.61;4.18]	4.14 [3.98;4.31]	0.25 [-0.08;0.58]			
Choice concerning vegetables	Pre	2.16 [1.81;2.51]	2.29 [2.09;2.50]	0.13 [-0.27;0.53]	5.10	0.026	0.041
	Post	2.58 [2.22;2.94]	2.30 [2.09;2.52]	-0.28 [-0.70;0.14]			
Modelling concerning fruit	Pre	4.16 [3.86;4.67]	4.33 [4.15;4.51]	0.17 [-0.18;0.53]	12.67	0.001	0.096
	Post	4.61 [4.34;4.89]	4.21 [4.05;4.37]	-0.40 [-0.72;-0.09]			
**Parental self-efficacy**							
SE giving an explanation concerning soft drinks	Pre	4.18 [3.86;4.50]	4.48 [4.30;4.67]	0.30 [-0.07;0.68]	5.23	0.024	0.044
	Post	4.43 [4.15;4.71]	4.31 [4.15;4.47]	-0.12 [-0.44;0.20]			
SE involving concerning vegetables	Pre	4.26 [3.91;4.61]	4.30 [4.10;4.50]	0.04 [-0.36;0.45]	5.88	0.017	0.046
	Post	4.58 [4.18;4.98]	4.10 [3.87;4.33]	-0.48 [-0.95;-0.02]			
SE motivating concerning fruit	Pre	3.55 [3.17;4.52]	3.78 [3.56;4.00]	0.23 [-0.21;0.67]	6.29	0.014	0.052
	Post	4.14 [3.75;4.52]	3.80 [3.58;4.03]	-0.33 [-0.78;0.11]			
SE involving concerning fruit	Pre	4.34 [4.04;4.65]	4.46 [4.29;4.64]	0.12 [-0.24;0.47]	4.76	0.031	0.039
	Post	4.62 [4.26;4.98]	4.26 [4.06;4.47]	-0.36 [-0.77;0.06]			

IG Intervention group; CG Control group; SE Self-efficacy; CI Confidence interval. ηp^2^ effect size; An effect size of 0.3 or lower is considered as small effect size. F-values, p-values and effect sizes were obtained by conducting repeated measures anova.

#### Evaluation of parents’ perception of the intervention

In total, 25 participants (80.6% of parents that received the entire “Movie Models” intervention) completed the questionnaire regarding parents’ perception of the integration of the videos and the format of the intervention.

In total, 44.0% of the parents indicated that the “Movie Models” videos were an important part of the Feel4Diabetes counselling sessions. Furthermore, 60.0% of the parents indicated that they applied tips regarding parenting practices that are covered in the videos and 52.0% of the parents indicated that the discussions regarding the videos were instructive for them. About half of the parents (52.0%) indicated that the discussions ensured that they applied tips in their own family. Finally, 36.0% of the participants indicated that they have talked with others (non-participants) about the videos and none of the parents (0%) watched the videos at home.

## Discussion

The present study examined the effect of integrating the “Movie Models” videos within the European Feel4Diabetes-intervention, on parenting practices and parental self-efficacy concerning parenting practices related to children’s PA, screen-time and eating behaviour in vulnerable families (i.e. families living in low SES municipalities and having an increased risk for developing T2D). Additionally, it was examined how the integration of the “Movie Models” videos and the intervention format was perceived by the participants.

Overall, the integration of the “Movie Models” videos within the Feel4Diabetes-intervention had some favourable effects on parenting-related factors regarding children’s health behaviours. After the intervention period, parents that received the entire “Movie Models” intervention are more likely to apply the following parenting practices: motivating their child to watch less TV or DVD, setting rules concerning the consumption of juice, letting their child choose between different kinds of vegetables and modelling fruit consumption of their child compared to the control group. In addition, parents assigned to the intervention group had an increase in self-efficacy concerning giving their child an explanation concerning soft drink, involving their child in buying vegetables and fruit and motivating their child to eat fruit. Although these intervention effects are statistically significant, the biological relevance should be interpreted with caution because the effect sizes are small (range: 0.039–0.096). Furthermore, no significant effects were found on most parenting practices (95% of parenting practices regarding PA and screen-time and 90.5% on parenting practices regarding eating behaviour) and related parental self-efficacy (95% of related parental self-efficacy regarding PA and screen-time and 89.2% of related self-efficacy regarding children’s eating behaviour). Because parenting-related factors are a bridging function between the intervention and children’s health behaviours, improving some parenting-related factors is a first important step in adopting children’s health behaviours.

The limited numbers of favourable intervention effects may be attributed to the fact that the intervention period (i.e. integration of the “Movie Models” videos within only two Feel4Diabetes counselling sessions) was too short to change most parenting-related factors. Furthermore, a larger number of effects were found on parenting-related factors regarding children’s eating behaviour compared to PA and screen-time, which is in line with the findings of De Lepeleere et al. (2017). This can possibly be explained by the fact that parenting practices and related parental self-efficacy regarding children’s eating behaviour are easier to integrate in families’ daily life because they eat at least three times a day. In addition, PA and screen-time might be seen as supplemental to the biological needs such as feeding [41]. Furthermore, the limited number of favourable intervention effects on parenting practices related to PA and screen-time can possibly be explained by the fact that parents of young children think that their child is already sufficiently active [55] and that parents considered educational exposure as a main benefit of watching TV [56]. Therefore, a first important step is to raise parents’ awareness about children’s unhealthy activity behaviours and about the related negative consequences for children’s health. However, this is just a hypothesis and needs further investigation.

When making the comparison between the pilot study “Movie Models” [41] and the integration of the “Movie Models” videos within the European Feel4Diabetes-study, two main differences exist. On the first hand, there is a clear difference in the format of delivering the intervention (online sessions vs. face-to-face group discussion sessions). Within the previous pilot study of the “Movie Models” intervention, the intervention group was invited to watch the “Movie Models” videos on an online secured website, individually at home [41]. Because literature indicated that vulnerable participants prefer a group based approach [57], and PA promotion programs focusing on vulnerable individuals including this approach achieved promising results [58], this study organized group sessions to deliver the “Movie Models” videos in a vulnerable group. During the group sessions, the videos were shown and afterwards discussed. Further, parents were asked to talk about child-parent situations that they personally perceived as difficult and possible appropriate reactions were then discussed afterwards. The group approach may have led to more attentive viewing of the videos and the discussion part might have caused that parents were more able to compare the videos with their current home situation and to reflect about it. Indeed, this face-to-face group discussion approach was relatively well received by the parents. Based on the evaluation of parents’ perception of the intervention, more than half of the parents applied tips regarding parenting practices that were covered in the videos, indicated that discussions regarding the videos were instructive for them and that the discussions ensured that they applied tips in their own family. Based on the results of the current study, the face-to-face group discussion approach used to integrate the “Movie Models” videos within the Feel4Diabetes intervention may be a promising way to improve some parenting practices and related parental self-efficacy regarding children’s health behaviours. The integration of “Movie Models” in the newly developed Feel4Diabetes-intervention was also well perceived by the researchers. The interventions were very compliant as they both aimed to inform parents regarding healthy behaviours and to improve these behaviours in their children. Further, “Movie Models” was integrated into Feel4Diabetes and not delivered as a separate intervention, to avoid confusion among the participants.

On the other hand, the pilot study focused on the general population, while the present study focused on vulnerable families, because literature showed that an unhealthy lifestyle is more prevalent in low SES municipalities and an unhealthy lifestyle also increases individuals’ risk for developing T2D [46, 59]. Furthermore, low educated parents have lower levels of favourable and higher levels of unfavourable parenting practices related to eating behaviour compared to higher educated parents [25]. Therefore, it was expected that the target group of this study had more room to improve their parenting practices and their related parental self-efficacy. Our sample had a mean FINDRISC-score of 12, which indicates a moderate risk for developing T2D (one in six individuals will develop T2D within 10 years) [46] and 28% of the included sample had a low family SES. Compared to the pilot study of “Movie Models” a higher percentage of families with low family SES could be reached [41]. So while targeting low SES municipalities resulted in reaching a higher percentage of low SES families and thus more families with an increased risk for developing T2D within the current study, we still mainly reached medium to high SES participants. Also in previous studies, researchers indicated that it is more difficult to recruit vulnerable groups [60] as they less often tend to participate in lifestyle interventions [61]. Future research should look for more effective strategies for identifying vulnerable groups. For example, contacting local health professionals, community workers or general practitioners working in low SES neighbourhoods to identify potential participants who might be eligible for participation or to involve them during the intervention [62, 63]. A greater impact on parenting-related factors would be expected when focusing only on a low individual SES population with a high risk for developing T2D.

A first strength of the current study is the cluster randomized design with a pre-test post-test evaluation. A second strength is the focus on vulnerable families. Although the recruitment remains a challenge, it is important to focus on this subgroup as they might be in greatest need for participation in this kind of lifestyle interventions [20, 25]. Furthermore, targeting T2D instead of overweight and obesity is a unique aspect and might be seen as a third strength within this study. Mentioning that the study targets T2D could have positively influenced the participation rate, for example in case of having a friend or member of the family been diagnosed with T2D or being aware of more severe complications related to this chronic disease, especially in vulnerable families. However, it remains unclear if the study target has indeed influenced the participation rate within the current study. This study also has some limitations. Firstly, by using self-reported questionnaires it may be possible that parenting practices and related parental self-efficacy were overestimated because of a social desirability bias. Second, volunteer bias may exist at the school and parent level. Volunteer bias at the school level can assumed to be limited since the participating and non-participating schools were all public and comparable in school size and area in which both schools were located. At the parent level, volunteer bias may have occurred during recruitment and retention. Throughout the Feel4Diabetes study, there is a very large drop in participants, which is considered as a major limitation of the current study. Further, by conducting per protocol analysis, even more people dropped out and attrition bias may exist. The results usually provide a lower level of evidence, but better reflect the effects of the intervention [64]. This might have influenced the outcomes of this intervention, so results should be interpreted with caution. Fourth, a low percentage of low individual SES participants could be reached by recruiting in low SES municipalities. So, a disadvantage of using area level SES as a measurement of SES could be an under or overestimation of SES [65]. Parental education is a more frequently used indicator to determine SES [65, 66]. It is relatively easy to measure in self-reported questionnaires, it has a high response rate, and is relevant to people regardless of age and working circumstances [67]. However, this indicator also has some limitations. Measuring the number of years of education contains no information about the quality of the education, which is important when linking education to health outcomes [67]. Moreover, within the current study, the significant intervention effects were attributed to the integration of the videos within the high-risk families component, however we cannot exclude the potential influence of the school- and community component on these variables. However, the significant intervention effects were mainly addressed on the more complex parenting-related factors, such as “how can I motivate my child to watch less TV or DVD” and no effects were found on the more concrete and obvious parenting practices [41]. Therefore, it seems more obvious that favourable intervention effects within the current study were attributed to the integration of the videos and not on the wider intervention. Finally, we now focused specifically on the effects of “Movie Models”, but further research is needed to investigate the broader effects of the Feel4Diabetes-intervention on children’s and adults’ health behaviours and other health-related factors (BMI, blood pressure, lipid profile, etc).

## Conclusion

The integration of the “Movie Models” videos within the European Feel4Diabetes-intervention showed no or limited effects on parenting practices and parental self-efficacy concerning parenting practices related to children’s health behaviours in vulnerable families. More parenting practices and related parental self-efficacy regarding children’s eating behaviour improved after the intervention period in comparison with PA and screen-time. In addition, the intervention was relatively well perceived by the parents. So, a face-to-face group discussion approach may be a promising way to improve parenting-related factors in vulnerable families. Further research is needed to find out which intervention strategies could enhance parenting practices and related parental self-efficacy regarding children’s PA and screen-time and to investigate if the Feel4Diabetes-intervention was effective in improving children’s health behaviours (i.e. levels of PA, SB (including screen-time) and eating behaviour).

## Supporting information

S1 Table Overview of intervention-effects on all parenting-related factors. (PDF)

S2 Table Consort Checklist. (DOC)

S1 File Study protocol of the Feel4Diabetes-study. (PDF)
