# Supplementary material for: Effect of integrating a video intervention on parenting practices and related parental self-efficacy regarding health behaviours within the Feel4Diabetes-study in Belgian primary schoolchildren from vulnerable families: A cluster randomized trial
Source: PLoS One. 2019 Dec 11;14(12):e0226131. doi: 10.1371/journal.pone.0226131 (PMC6905545; doi:10.1371/journal.pone.0226131)
Supplement: S1 Table — (PDF) [file pone.0226131.s001.pdf]

S1 Table 1. Overview of intervention effects on all parenting-related factors

**Per protocol analysis**<br>Control n=95<br>Intervention n=31		
PHYSICAL ACTIVITY AND SCREEN-TIME		
Parental practices related to children's physical activity and screen time		
	F-value	p-value
Availability of sports material	1.130	0.290
Monitoring PA	0.003	0.956
Modeling concerning PA	0.008	0.929
Motivating concerning PA	0.508	0.478
Reinforcing concerning PA	2.464	0.119
Giving choice concerning PA	0.000	0.983
Involving in sport	1.203	0.275
Involving in household chores	1.091	0.298
Rules concerning TV or DVD	0.465	0.496
Rules concerning games	0.341	0.561
Being consistent concerning TV or DVD	0.006	0.939
Being consistent concerning games	1.852	0.177
Giving an explanation concerning TV or DVD	0.560	0.456
Giving an explanation concerning games	0.324	0.571
Monitoring watching TV or DVD	2.126	0.147
Monitoring gaming	1.494	0.224
Modeling concerning TV or DVD	2.337	0.129
Modeling concerning PC	0.403	0.527
Motivating concerning TV or DVD	8.487	**0.004****
Motivating concerning PC	0.196	0.659
Parental self-efficacy in parental practices related to children's physical activity and screen time		
	F-value	p-value
SE Availability of sports material	0.415	0.520
SE Monitoring PA	0.111	0.739
SE Modeling concerning PA	1.762	0.187
SE Motivating concerning PA	0.256	0.614
SE Reinforcing concerning PA	0.640	0.425
SE Giving choice concerning PA	0.000	0.993
SE Involving in sport	0.013	0.911
SE Involving in household chores	0.040	0.843
SE Rules concerning TV	0.606	0.438
SE Rules concerning games	0.658	0.419
SE Being consistent concerning TV	0.000	0.997
SE Being consistent concerning gaming	1.269	0.263
SE Giving an explanation concerning TV	**4.539**	**0.035****
SE Giving an explanation concerning games	0.255	0.614
SE monitoring watching TV	0.429	0.514
SE Monitoring gaming	0.076	0.783
SE Modeling concerning TV	0.570	0.452
SE Modeling concerning PC	0.002	0.967

SE Motivating concerning TV	0.705	0.403
SE Motivating concerning PC	0.224	0.637
NUTRITION INTAKE		
Parental practices related to children's eating behaviour		
	F-value	p-value
Rules concerning soft drinks	0.226	0.636
Being consistent concerning soft drinks	0.276	0.601
Giving an explanation concerning soft drinks	0.777	0.380
Monitoring soft drink consumption	0.032	0.858
Modeling concerning soft drinks	0.976	0.325
Motivating concerning soft drinks	1.251	0.266
Availability of soft drinks	2.101	0.150
Rules concerning juice	5.216	**0.024***
Being consistent concerning juice	1.440	*0.233*
Giving an explanation concerning juice	0.045	0.832
Monitoring juice consumption	0.838	0.362
Modeling concerning juice	1.176	0.280
Motivating concerning juice	0.091	0.764
Availability of juice	2.112	0.149
Rules concerning snacking	0.020	0.887
Being consistent concerning snacks	0.334	0.564
Giving an explanation concerning snacks	0.102	0.750
Monitoring snack consumption	0.000	0.984
Modeling concerning snacks	3.120	0.080
Motivating concerning snacks	0.263	0.609
Availability of snacks	6.992	**0.009****
Monitoring vegetable consumption	0.000	0.992
Modeling concerning vegetables	0.366	0.546
Motivating concerning vegetables	0.037	0.847
Reinforcing concerning vegetables	0.864	0.354
Choice concerning vegetables	5.104	**0.026***
Availability of vegetables	0.424	0.516
Involving concerning vegetables	0.702	0.404
Monitoring fruit consumption	0.035	0.853
Modeling concerning fruit	12.666	**0.001****
Motivating concerning fruit	0.337	0.563
Reinforcing concerning fruit	2.554	0.113
Availability of fruit	3.369	0.069
Choice concerning fruit	0.324	0.338
Involving concerning fruit	1.255	0.265
Monitoring water consumption	0.312	0.578
Modeling concerning water	1.200	0.276
Motivating water consumption	0.075	0.785
Reinforcing concerning water	1.944	0.166
Choice concerning water	0.279	0.598
Obligating empty plate	2.102	0.150
Obligating to taste	1.674	0.198
Parental self-efficacy in parental practices related to children's nutrition intake		

Table 3 1Overview

Table 3 2S1 Table 1. Overview of intervention-effects on all parenting-related factors.

	F-value	p-value
SE Rules concerning soft drinks	0.148	0.701
SE Being consistent concerning soft drinks	2.576	0.111
SE Giving an explanation concerning soft drinks	5.230	**0.024****
SE Monitoring soft drink consumption	0.339	0.561
SE Modeling concerning soft drinks	0.377	0.540
SE Motivating concerning soft drinks	3.193	0.077
SE Rules concerning juice	3.615	0.060
SE Being consistent concerning juice	0.002	0.969
SE Giving an explanation concerning juice	3.625	0.060
SE Monitoring juice consumption	0.465	0.497
SE Modeling concerning juice	1.235	0.269
SE Motivating concerning juice	0.059	0.809
SE Rules concerning snacks	0.001	0.973
SE Being consistent concerning snacks	2.450	0.120
SE Giving an explanation concerning snacks	3.256	0.074
SE Monitoring snack consumption	0.185	0.668
SE Modeling concerning snacks	1.818	0.180
SE Motivating concerning snacks	1.843	0.177
SE Monitoring vegetable consumption	0.149	0.700
SE Modeling concerning vegetables	0.883	0.349
SE Motivating concerning vegetables	0.966	0.328
SE Reinforcing concerning vegetables	3.345	0.070
SE Choice concerning vegetables	0.689	0.408
SE Availability of vegetables	0.238	0.626
SE Involving concerning vegetables	5.881	**0.017***
SE Monitoring fruit consumption	0.835	0.363
SE Modeling concerning fruit	2.414	0.123
SE Motivating concerning fruit	6.293	**0.014***
SE Reinforcing concerning fruit	2.914	0.091
SE Choice concerning fruit	0.044	0.835
SE Availability conserning fruit	2.174	0.143
SE Involving concerning fruit	4.763	**0.031***
SE Monitoring water consumption	0.413	0.522
SE Modeling concerning water	0.034	0.853
SE Motivating concerning water	0.066	0.798
SE Reinforcing concerning water	0.618	0.434
SE Choice concerning water	1.619	0.206

SE self-efficacy

* borderline significant p-values (p≤0.05)

** significant p values (p≤0.01)
