# Supplementary material for: Effect of integrating a video intervention on parenting practices and related parental self-efficacy regarding health behaviours within the Feel4Diabetes-study in Belgian primary schoolchildren from vulnerable families: A cluster randomized trial
Source: PLoS One. 2019 Dec 11;14(12):e0226131. doi: 10.1371/journal.pone.0226131 (PMC6905545; doi:10.1371/journal.pone.0226131)

FEEL4DIABETES

Work Package 6

Development, implementation and evaluation of the intervention

Deliverable 6.1
HUA

Structured protocol and timeframe of implementation for the two components of the intervention and a protocol for the multistage randomization, the implementation and the evaluation of the two components of the intervention developed in English

1. Overview of the Feel4Diabetes-intervention

The Feel4Diabetes-intervention will be implemented in six European countries, namely Belgium, Bulgaria, Finland, Greece, Hungary and Spain. More specifically, based on the findings of Work Package (WP) 2, the intervention will be applied in the general population in Bulgaria and Hungary and in low-socioeconomic areas in the rest of the countries. In all countries, the families at high risk for developing type 2 diabetes will be identified through an algorithm which is based on the FINDRISC-questionnaire and was developed in WP5 (WP5 – deliverable 5.1).

The intervention will consist of two components: the “targeting all families” component which will be delivered at schools and the “targeting high-risk families” component which will be delivered out of the school setting, in families found to be at increased risk for type 2 diabetes according to the WP5-algorithm. The development of both components will be guided by the outcomes of WPs 2-4 regarding the barriers and facilitators of the behaviours found to be associated with risk factors for developing type 2 diabetes, existing legislation and policies, available human resources and infrastructure for both implementing the intervention as well as providing easy access to facilities for leisure time physical activity, active commuting etc.

Figure 1. Feel4Diabetes-intervention design

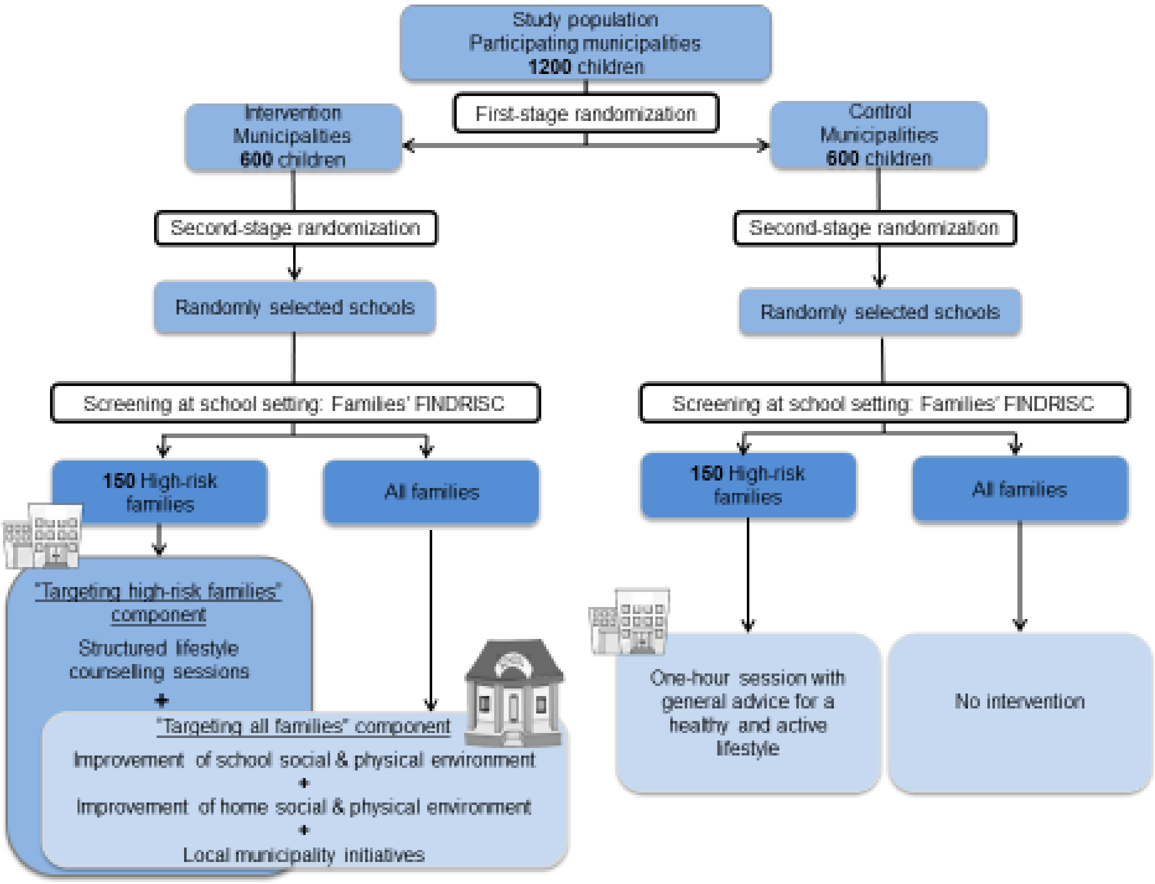

2. Ethical approvals

Approvals from the local authorities (Ethical Committee, National Ministry of Education, Municipality, etc.) will be taken in each intervention country before the start of the baseline measurements. Participants (parents/caregivers) will be asked to return a signed consent form for the participation of themselves and their children before the baseline measurements.

The Feel4Diabetes-intervention has been registered in the https://clinicaltrials.gov/ with the following registration number: NCT02393872.

3. Sampling procedure

3.1 Municipality, school district or equivalent unit recruitment

To identify the municipalities/school districts/or other equivalent units where Feel4Diabetes will take place we need to follow the next steps:

Step 1. Create tertiles of socioeconomic (SES) groups regarding the municipalities/school districts/or other equivalent units, using any existing/available socioeconomic data/variables in your country. In the case of Belgium, Finland, Greece and Spain municipalities/school districts/or other equivalent units will be recruited only from low-SES areas as described in the next steps below. In the case of Bulgaria and Hungary municipalities/school districts/or other equivalent units will be recruited from any area of these countries.

Step 2. Among the low-SES groups randomize the municipalities/school districts/or other equivalent units to intervention and control group to achieve a 1:1 ratio in the number of children in each intervention arm.

Step 3. Apply/get approval from all local authorities (Ethical Committees, Ministries, Municipalities etc.) for the randomly selected municipalities/school districts/or other equivalent units.

Step 4. Create lists of all schools within the randomly selected municipalities/school districts/or other equivalent units. Proceed with the random selection of schools from these lists in each municipality until you reach the recruitment goal of 1500 children/families for the “all families component” and 360 children/families for the “high-risk component” of the Feel4Diabetes-intervention.

All intervention countries should ensure that there will be a reasonable distance between the intervention and the control municipalities, in order to minimize contamination between the intervention and the control group.

3.2 Contacting schools and families

To increase the participation rate of schools and families (parents/caregivers), the following procedures could be followed when approaching schools for recruitment (but this may vary according to local and country situations):

- School/Individual benefits: It is important to explain to headmasters and/or teachers the aim of the programme and how important their active participation and contribution is in developing EU future public health policies and health promotion activities aiming to promote health and well-being of children in Europe. Also, explain benefits that both schools and children will get out of this procedure. Such benefits for the schools can be the use of the intervention material in their educational programme.
- Confidential information: It is important to emphasize to headmasters and/or teachers and parents that all school results and information received during the evaluation period will remain strictly confidential.
- Communication with schools: All telephone contacts with headmasters and/or teachers should be clear and friendly. A clear explanation of the aim of the programme should be provided as well as specific answers to possible questions. Make sure that there is a well oriented, knowledgeable on the topic and available individual to always answer the phone on the contact number you have provided and handle e-mail or fax communication.

Once a positive response is received, arrange a meeting in order to provide more information on the programme and on its next steps. Make separate printouts on original letterhead paper and sign each letter by hand including the name of the headmaster and the school in the letter, instead of making one standard letter. Carry the information letter and the consent form with you in the meeting and make sure you are well prepared and organized to provide them with all necessary documents.

- Parents'/caregivers' information letter and consent form: Following the acceptance of the school and the teacher(s) to participate in the programme, parents' information letters and consent forms for the parents and the child should be circulated by the teachers to obtain the parents'/caregivers' approval. Do the best you can to gain the trust and enthusiastic collaboration of the teachers to ensure that they will become our allies and active partners in the programme. More specifically, it is essential that teachers act as "ambassadors" of the programme to the parents/caregivers, distribute the consent forms and, if approved by the country ethical committee, gather their approvals. Thus, teachers will have a supportive role in encouraging parents/caregivers to take part in the programme. Teachers and researchers will also be in collaboration in order to collect the filled in consent forms. The researcher will make a call to the school the day before picking up the consent forms, so that the teacher will give

a last reminder to parents of children that want to participate to bring it back filled in. However, if this is not possible (for example, if due to needs for data protection teachers cannot collect data on children and parents), questionnaires and consent forms may be collected e.g. by mail.

- Numbers of schools contacted and participation rate of schools and parents: should be continuously recorded and updated during the recruitment phase.

3.3 Eligibility criteria

Schools are considered eligible if they fulfill the following criteria:

1. The headmaster and the teacher(s) have agreed their school to participate.

Children/families are considered eligible if they fulfill the following criteria:

1. Children attend the first grades of compulsory education at the time of baseline measurements.
2. Parents/caregivers provided a consent form for themselves and their children.

4. Intervention

The Feel4Diabetes-intervention includes two components:

A) The "targeting all families" component

The "targeting all families" component will be implemented in all schools by teachers targeting all families and will run for two academic years. The first year will be targeting changes at the school, family and neighborhood environment, family involvement and behavioural modification, while the second year will have a follow-up intervention aiming to maintain the changes achieved during the first year.

Teachers of the intervention group will be invited to a training session (about 2 hours), which will take place before the start of the intervention. The main goal of this training is to train them on how to deliver the intervention. Of equal importance is to create a team spirit with the teachers and get to know each other. The venue for the training will be easily accessible and as close as possible to the intervention districts (e.g. school, university, municipality or any other setting) and will be chosen by each local team. Standard PowerPoint-presentations will be developed in English by the WP6-leader (Harokopio University Athens), translated in the six local languages and adapted according to the local needs/conditions/regulations by each intervention country.

The teachers training will be repeated before the start of the second year of the intervention, in order to train the new teachers.

B) The “targeting high risk families” component

The “targeting high risk families” component comprises of 7 counseling sessions, spread over one school year, in which adult members of the families will be invited to attend. These sessions will be implemented in health promotion centers or other locally available facilities (e.g. school, university, municipality or any other setting) easily accessible, friendly and as close as possible to the intervention districts by members of each partner’s research team. Each partner needs to contact the authorities of these centers/facilities to ensure that they will be available/accessible for the delivery of the counseling sessions during the first year of the intervention.

Mobile text messages will be used in order to increase motivation and adherence to the recommendations using SMART (specific, measurable, achievable, relevant, and timely) goals during the second year of the intervention.

In order to ensure comparability among countries and researchers: (i) the same number of sessions will be offered, (ii) the same behavioural change theories and motivational tools will be applied, and (iii) the same recommendations regarding a healthy diet and active lifestyle will be targeted. However, the means by which these recommendations are to be reached as well as the motivational triggers used will be tailor made not only at a country level, but also at family as well as individual level. This tailor made approach will be followed during the first year of the intervention via the counseling sessions, as well as during the second year of the intervention via text messages.

The current protocol and any other relevant material (handbook and presentation for teachers, newsletters for parents, manual of operation for researchers, diaries and/or leaflets for the “targeting high risk families” component, etc.) for the teachers and families at school and for the high-risk families in the counseling sessions, which will be developed in WP6 will be used in the implementation of the intervention.

5. Impact and outcome evaluation

All measurements which will be conducted to assess the impact and outcome of the Feel4Diabetes-intervention will take place at baseline (2016), follow-up 1 (2017) and follow-up 2 (2018).

“Targeting all families” component

The evaluation of the “targeting all families” component will be based on the following:

a) *Measurement of children’s anthropometric indices*: specifically children’s weight and height will be measured by the research team. Researchers from all intervention countries (at least one per country) were centrally trained in

the 2nd Feel4Diabetes-meeting in Ghent, Belgium (September 2015) in order to conduct the measurements as described in the WP5-protocol. These researchers will locally train the rest of researchers who will be involved in each intervention country (following the same procedure as in Ghent to ensure that the same level of intra- and inter-observer reliability is achieved within the members of each local research team). For more information regarding the protocol for the anthropometric measurements, please see the relevant protocol (WP5).

b) *Assessment of FINDRISC-score of at least one or preferably two parents/stepparents:* via the FINDRISC-questionnaire (WP5).

c) *Assessment of child's and his/her parent's or primary caregiver's energy balance related behaviours and their determinants:* via a short questionnaire (WP5) which includes questions on family's targeted energy balance related behaviours and their determinants, which will be completed by the child's parent or primary caregiver.

"Targeting high risk families" component

From each one of the parents and grandparents (if they attend the baseline and follow-up 1 and 2 measurements) the following data will be collected:

a) *Anthropometric indices:* specifically adults' weight, height and waist circumference will be measured by the research team.

b) *Blood pressure:* specifically adults' systolic and diastolic pressure will be measured by the research team.

c) *Blood samples:* samples will be collected by the research team (doctor/nurse) early in the morning after overnight fasting. Blood processing procedures and analyses will be performed locally and fasting glucose, insulin, total and HDL cholesterol as well as triglycerides will be analyzed following a standardized procedure to ensure comparability in all countries.
In each intervention center, the blood analyses will be performed by one laboratory, which will be certified and will be the same in all evaluation phases (baseline, follow-up 1 and follow-up 2), following exactly the same procedures.

d) *Assessment of food intake and eating behaviours:* via food frequency questionnaire (FFQ), aiming to record food intake, and eating behaviour questionnaire, aiming to obtain information regarding social and physical environment determinants of eating behaviours and sub-behaviours.

e) *Assessment of physical activity levels and related behaviours via:*
   i. Pedometers
   ii. Physical activity/ sedentary behaviours questionnaire aiming to obtain information regarding social and physical environment determinants of physical activity/ sedentary behaviours and sub-behaviours.

Regarding the children from the high-risk families the data to be obtained beyond those that will be obtained from the “all families” component will be the assessment of food intake and eating behaviour and physical activity levels and related behaviours via:

i. Food frequency questionnaire (FFQ), aiming to record food intake, and eating behaviour questionnaire, aiming to obtain information regarding social and physical environment determinants of eating behaviours and sub-behaviours.
ii. Pedometers
iii. Physical activity/ sedentary behaviours questionnaire aiming to obtain information regarding social and physical environment determinants of physical activity/ sedentary behaviours and sub-behaviours.

For more information regarding the protocol for the anthropometric, physical activity (pedometers) and blood pressure measurements and regarding the blood sampling/analysis procedures, please see the relevant protocol (WP5).
The results of children’s and parents’ weight status and physical activity levels (pedometers) and the results of the blood analysis and blood pressure will be delivered personally and confidentially in a sealed envelope to each family during the first session.

6. Training of researchers to conduct the measurements and deliver the intervention (both components)

A training of the fieldwork researchers was conducted in December 3-4, 2015, in Budapest, Hungary. During this meeting, the following topics were discussed/ covered and fieldwork researchers were centrally trained and familiarized on how to do the tasks below:

1. Ethical approvals: the countries that had not taken the approvals by the time of the meeting, informed the group on potential barriers/problems, in order to find solutions and set deadlines for taking these approvals.
2. Approaching and getting approvals to enter the schools.
3. Gaining trust and enthusiastic collaboration of school headmasters and teachers.
4. Getting consent forms from the parents for the school-based intervention.
5. Applying measurements and collecting/entering data.
6. Identification of high-risk families, using the WP5-algorithm.
7. Invitation of high-risk families and getting their consent to undergo the additional measurements described above.

8. Applying measurements to the high-risk families and collecting/entering data.

Central trainings of the researchers who will train the teachers in the school-based ("targeting all families") component of the intervention and/or the researchers who will deliver the counseling sessions to the out-of-school ("targeting high-risk families") component of the intervention have been planned in February 16-17, 2016 (Helsinki, Finland) and in August 2016 (Sofia, Bulgaria).

7. Process evaluation and cost-effectiveness

- **Process evaluation** is a procedure needed to assess the level of compliance of the study participants (e.g. teachers, researchers delivering the counseling sessions, etc.) with the study protocol.
  For more information regarding the process evaluation plan and tools, please see the relevant document (WP6 – deliverable 6.5).
- **Cost-effectiveness** is a procedure needed to assess whether the amount of money spent for the delivery of the intervention in each country resulted in lifestyle changes which can prospectively save money for the health system.
  For more information regarding the cost-effectiveness tools, please see the relevant questionnaires (WP7 – deliverable 7.2).

8. Timeframe of intervention (development, recruitment, implementation and evaluation)

Figure 2. Timeframe of the Feel4Diabetes-intervention

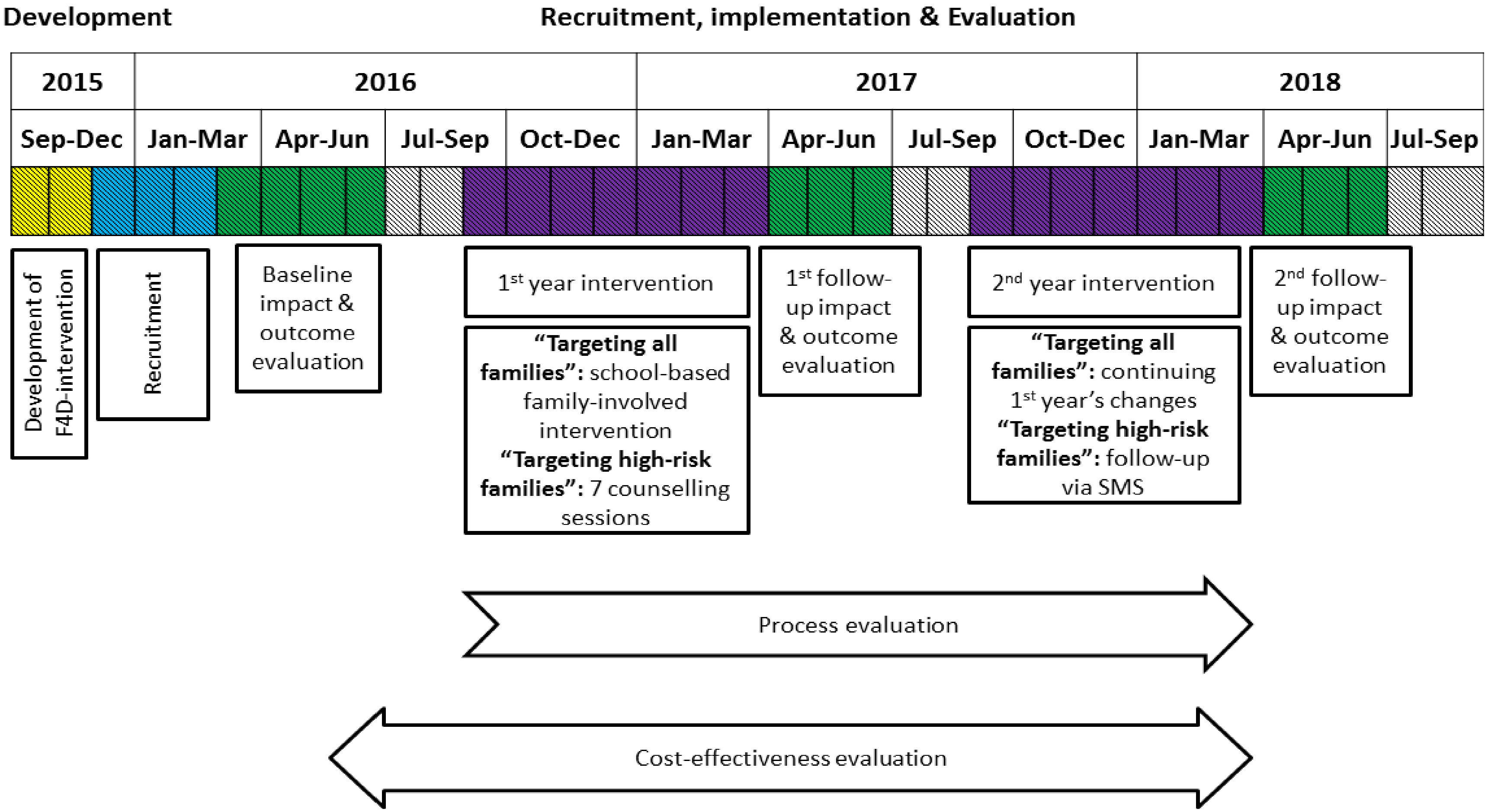

Supplement: S1 File — (PDF) [file pone.0226131.s003.pdf]
